# A Genetic Screen to Discover Pathways Affecting Cohesin Function in *Schizosaccharomyces pombe* Identifies Chromatin Effectors

**DOI:** 10.1534/g3.112.003327

**Published:** 2012-10-01

**Authors:** Zhiming Chen, Scott McCroskey, Weichao Guo, Hua Li, Jennifer L. Gerton

**Affiliations:** * Stowers Institute for Medical Research, Kansas City, Missouri 64110, and; † Department of Biochemistry and Molecular Biology, University of Kansas Medical Center, Kansas City, Kansas 66160

## Abstract

Cohesion, the force that holds sister chromatids together from the time of DNA replication until separation at the metaphase to anaphase transition, is mediated by the cohesin complex. This complex is also involved in DNA damage repair, chromosomes condensation, and gene regulation. To learn more about the cellular functions of cohesin, we conducted a genetic screen in *Schizosaccharomyces pombe* with two different cohesin mutants (*eso1*-G799D and *mis4*-242). We found synthetic negative interactions with deletions of genes involved in DNA replication and heterochromatin formation. We also found a few gene deletions that rescued the growth of *eso1*-G799D at the nonpermissive temperature, and these genes partially rescue the lagging chromosome phenotype. These genes are all chromatin effectors. Overall, our screen revealed an intimate association between cohesin and chromatin.

The generation of cohesion between sister chromatids takes place during DNA replication and dissolves at the metaphase to anaphase transition. Cohesion allows sister chromatids to biorient on the mitotic spindle and segregate accurately when the cell divides. Cohesion is mediated by the cohesin complex in cooperation with additional factors. In addition to its essential role in chromosome segregation, cohesin plays roles in chromosome condensation, DNA damage repair, and gene regulation. The role in gene regulation has been proposed to occur through several different mechanisms, including cohesin promoting gene looping, barrier function, enhancer−promoter interactions, and RNA pol II elongation (Dorsett 2011).

The generation of cohesion is dependent on an acetyltransferase that acetylates the Smc3 subunit of the cohesin ring to stabilize cohesion (Rolef Ben-Shahar *et al.* 2008; Unal *et al.* 2008; Zhang *et al.* 2008). This acetyltransferase is known as Eco1 in budding yeast, Eso1 in *S. pombe*, and ESCO2 in mammals. Mutations in both copies of ESCO2 are associated with the human disease Roberts syndrome (RBS) (Vega *et al.* 2005). Most of the mutations are missense mutations in which case ESCO2 protein is not detected, but a mutation that affects the active site also has been identified in association with RBS (W539G) (Vega *et al.* 2005). One hallmark of metaphase chromosomes in RBS is that they show “heterochromatic repulsion,” which refers to regions of “puffing” at heterochromatic regions around the centromeres and nucleolar organizers (Schule *et al.* 2005). Heterochromatin has been shown to be important for cohesin binding at pericentric regions in *Schizosaccharomyces pombe* (Bernard *et al.* 2001; Nonaka *et al.* 2002). Cohesin also associates with many locations in chromosome arms (Schmidt *et al.* 2009).

Eso1p in *S. pombe* acetylates evolutionarily conserved lysine residues in Psm3, in a process that appears to be similar to that reported in *S. cerevisiae* and humans. Acetylation is critical for the establishment of cohesion during DNA replication in both mitosis and meiosis (Feytout *et al.* 2011; Kagami *et al.* 2011). Mutation of both lysine residues in Psm3 to the acetyl-mimicking asparagine makes *eso1* dispensable, although surprisingly the nonacetylatable mutant also was viable but did have cohesion defects (Feytout *et al.* 2011). *eso1* in *S. pombe* is actually a fusion of two genes that are separate in *S. cerevisiae* and mammals. The N-terminal two-thirds is homologous to *RAD30*, also known as DNA polymerase eta, which is involved in translesion synthesis during postreplication DNA repair (Tanaka *et al.* 2000; Madril *et al.* 2001). The C-terminal one-third is homologous to *ECO1*. The *ECO1* domain is sufficient for the establishment of cohesion in *S. pombe* because deletion of the N-terminus increases sensitivity to ultraviolet irradiation but does not compromise cohesion (Tanaka *et al.* 2000). All these data suggest the importance of acetylation activity in *S. pombe* and the evolutionarily conserved function of *eso1* in cohesion establishment.

Given the many functions of cohesin, we decided to conduct an unbiased genetic screen to identify gene deletions that would act synthetically with an allele of *eso1*. The results of the screen could help highlight the roles of cohesin in various aspects of chromosome metabolism. We used a query strain bearing a mutation in the acetyltransferase domain of *eso1* that compromises the catalytic activity of the protein (*eso1*-G799D, originally *eso1*-H17). The *eso1*-G799D mutation confers sensitivity to elevated growth temperature (Tanaka *et al.* 2000). We chose to conduct the screen in *S. pombe* because 1) there is a collection of 3066 strains with deletions in the nonessential genes (Kim *et al.* 2010) and 2) *S. pombe* displays heterochromatic properties similar to higher eukaryotes. Our screen identified gene deletions that in combination with *eso1*-G799D had (1) negative effects on growth (synthetic sick) and (2) rescued growth at nonpermissive temperature (synthetic rescue). One of the major gene classes with negative effects were genes involved in heterochromatin function. These genes also displayed synthetic negative interactions with a second cohesin allele, *mis4*-242. *mis4* is involved in cohesin loading (Tomonaga *et al.* 2000). We identified and verified three new deletions that partially rescued the growth of *eso1*-G799D at elevated temperatures, all of which are genes whose protein products operate on chromatin. Overall, our findings suggest an intimate relationship between cohesin and chromatin.

## Materials and Methods

### Strains and media

All the strains used in this study are listed in supporting information, Table S6. The culture media used for *S. pombe* was YES except where otherwise stated. *S. pombe* strains were grown at 32°, except that the temperature sensitive strains were grown at 25°. For serial dilution plating assays, 10-fold dilutions of a log-phase culture were plated on the indicated medium and grown for 3 to 4 days. Thiabendazole (10 μg/mL) was used for the sensitivity test. For silencing assays, the strains with *ura4+* reporter gene inserted at outer repeat region of centromere1 (otr1::*ura4+*) were used. Serial dilutions of the wild-type and respective mutants were plated on YES, and YES plates containing FOA. DAPI staining were used to determine the percentage of lagging chromosomes as described previously (Gregan *et al.* 2007).

### Mutagenesis and gene disruption

To construct the *eso1* mutant strains, the C-terminus of *eso1* was amplified from genomic DNA and cloned into Pclonat1 (a gift from Gregan’s laboratory). The construct was subjected to site-directed mutagenesis. Plasmids carrying mutated *eso1* were linearized with *Mfe*I and transformed into the PEM2 *S. pombe* strain. Positive transformations were identified by polymerase chain reaction, and point mutation was verified by sequencing. The 3066 G418-resistant, haploid single-deletion mutants were obtained from the BIONEER (V 2.0). To make gene deletion strains, each individual gene deletion cassette was amplified from the genomic DNA of BIONEER gene deletion collection. The forward and reverse primers are designed about 250 bp upstream or 150 bp downstream of the open reading frame. After transformation, proper integration of the KanMX1 cassette in positive colonies was verified by colony polymerase chain reaction. Transformation was conducted with the lithium acetate method as described previously (Gregan *et al.* 2006).

### Genetic crosses

Genetic crosses were performed according to the PEM procedure as described previously (Roguev *et al.* 2007, 2008). In summary, a PEM2 strain with either the *eso1*-G799D or *mis4*-242 mutation was used as the query strain to cross against the whole gene deletion library. Taking advantage of the background of PEM2 strains, after mating and sporulation, we used cycloheximide to select against the unsporulated diploid cells and h+ haploid cells. The mutant *eso1* and *mis4* genes of the query strain were fused with the NatMX cassette, which confers the resistance to nourseothricin (aka clonNAT), and the test strain from deletion collection has anti-G418 background; therefore, 100 μg/mL G418 and clonNAT was used to select the double mutants after haploid selection. Images of the agar plates were analyzed and processed. The plates with and without treatment of clonNAT were set as control and test plates, respectively. The *eso1* screen was performed twice, with either 4 or 12 individual spots scored for growth. The *mis4* screen was performed once with four individual spots scored for growth. All primary data for this article can be found at http://srdr.stowers.org/websimr/datasetview/474/0/.

### Data processing and quality assessment

Images of the agar plates were acquired and analyzed. We normalized the colony sizes to correct for differences in growth conditions(Collins *et al.* 2006). In summary, the colony sizes of the outermost two rows and two columns are normalized to their plate middle mean, and then the colony sizes on each plate were scaled such that the middle means for all plates were equal a fixed number, which was the median of plate middle means across all plates. We used paired *t*-test to compare the average colony size of double mutants to single mutants and recorded t-statistics and p-values for each test. We combined *P* values from two independent experiments by using the Fisher method whenever possible.

## Results

### Characterization of cohesin alleles in *S. pombe*

**Figure 1:**
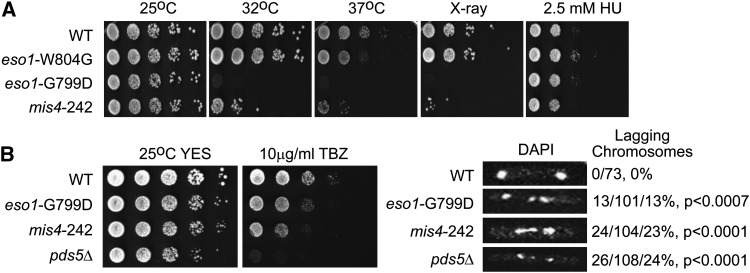
Basic characterization of cohesin alleles in *S. pombe*. (A) Cohesin mutants were grown at 25°, 32°, and 37°. *eso1*-G799D and *mis4*-242 mutants can grow at 25° but not at greater temperatures, and both of these mutants are sensitive to 75-Gy X-ray treatment. *eso1*-W804G, an analogous mutation to that associated with RBS, grew normally at 32° and grew only slightly slower at 37°. The *eso1*-W804G mutant did not show a significant growth defect upon exposure to X-rays. None of the mutants were hypersensitive to 2.5 mM hydroxyurea (HU). (B) TBZ sensitivity was tested, and lagging chromosomes were scored. *P* values are derived from a Fisher test using wild-type (WT) as the reference.

To choose alleles for genetic screening, we compared the behavior of different cohesin mutations. *eso1*-G799D has been previously reported to exhibit temperature-sensitive growth and defects in double-strand DNA break repair (Tanaka *et al.* 2000). This allele is analogous to the *eco1-1* allele in budding yeast, which severely compromises acetyltransferase activity. We compared the behavior of an *eso1*-G799D mutant strain with one bearing *eso1*-W804G, which is analogous to a mutation associated with RBS and also compromises acetyltransferase activity (Vega *et al.* 2005). We also evaluated a mutation in Mis4/Scc2, *mis4*-G3965A(*mis4*-242), a previously reported temperature sensitive mutation (Tanaka *et al.* 2000; Toyoda *et al.* 2002). Mis4 is part of a cohesin loading complex (Furuya *et al.* 1998; Tomonaga *et al.* 2000). Mutations in *mis4* are associated with Cornelia de Lange syndrome in humans (Tonkin *et al.* 2004). All mutant genes were expressed from the native promoter at their endogenous locus. When we grew these strains at 25°, 32°, and 37°, both *eso1*-G799D and *mis4*-242 were dead at 37° (Figure 1). A strain with *eso1*-W804G grew normally at 32°, and even a bit at 37°, although it was slow relative to a wild-type strain. Both the *eso1*-G799D and *mis4*-242 strains were sensitive to X-rays (Figure 1), consistent with a role for cohesin in double-strand DNA break repair (Furuya *et al.* 1998; Tanaka *et al.* 2000). The *eso1*-W804G strain did not show a significant growth defect upon exposure to X-rays. None of the mutants were hypersensitive to 2.5 mM hydroxyurea, a drug that will slow DNA replication (Figure 1). The *eso1*-W804G mutant seems to show a weaker phenotype compared with *eso1*-G799D, similar to what has been observed in budding yeast (Lu *et al.* 2010).

We further checked whether *eso1*-G799D showed phenotypes consistent with defects in chromosome segregation at 25° (Figure 1B). *eso1*-G799D and *mis4*-242 mutants were sensitive to thiabendazole (TBZ), a microtubule depolymerizing agent. Strains with defects in chromosome segregation are often sensitive to this agent. The *pds5Δ* mutant was extremely sensitive to TBZ. *pds5* contributes to cohesion maintenance in *S. cerevisiae* (Hartman *et al.* 2000) but is not essential in *S. pombe* (Tanaka *et al.* 2001). Pds5, along with Rad61/Wpl1, may also function as negative regulators of cohesion (Rolef Ben-Shahar *et al.* 2008; Heidinger-Pauli *et al.* 2009; Rowland *et al.* 2009; Sutani *et al.* 2009; Feytout *et al.* 2011). We used DAPI staining to monitor lagging chromosomes. All the cohesin mutants had some lagging chromosomes. Overall, these results suggest that chromosome segregation is affected by the cohesin mutants at the permissive temperature.

### A genetic screen with the eso1-G799D allele

To explore genetic interactions, we created a query strain with the *eso1*-G799D mutation in the PEM2 background (Roguev *et al.* 2008). Since the *eso1*-G799D did not grow at 37°, we could easily screen for suppressor mutations. We crossed the *eso1*-G799D strain against the whole gene deletion collection from BIONEER and followed published protocols to obtain double mutants (Roguev *et al.* 2007). The double mutants were plated at 25° to examine synthetic interactions and 37° to discover deletions that would rescue the temperature sensitivity. Colony size was used as a quantitative phenotypic readout. We used paired *t*-test to compare the average colony size from test plates to control plates. Genes within 500 kb of *eso1* or *rpl44* (a gene used as part of the selection procedure) were eliminated from the analysis as the result of linkage (Roguev *et al.* 2008). After elimination of linkage bias, a normal distribution of t-statistics was observed.

We found 215 genes that had a significant negative interaction with the *eso1*-G799D mutation by statistical analysis (Adjusted *P* < 0.05 and average difference of colony size >25; Table S1). Furthermore, we identified several genes whose deletion rescued growth of *eso1*-G799D at 37°. Many of the genes with negative interactions function in heterochromatin formation. To further confirm the negative interaction, we randomly selected several genes from these 215 genes. Serial dilution growth assay were performed, and 23 gene deletions were confirmed to be synthetically sick with the *eso1*-G799D mutation (Figure S1 and Table S2). Gene ontology term analysis regarding these 215 genes was conducted, and several GO categories are highly overrepresented in our synthetic sick gene list. Consistent with the fundamental role of *eso1* in cohesion establishment and chromosome segregation, GO terms of chromosome segregation, and cell cycle are highly overrepresented (Table S4). Interestingly, we also found that the GO term “gene silencing” was overrepresented.

Eco1 normally establishes cohesion during DNA replication at S phase, and accumulating evidence indicates the strong connection between cohesion establishment and DNA replication (Skibbens *et al.* 1999; Kenna and Skibbens 2003; Skibbens 2004; Moldovan *et al.* 2006). Moreover, mutations that affect DNA replication also cause cohesion defects both in *S. pombe* and *S. cerevisiae* (Skibbens 2004; Ansbach *et al.* 2008). We found that deletion of *ctf8*, *chl1*, or *swi3* showed synthetic negative effects with the *eso1*-G799D mutation. Ctf8 is part of an alternative replication factor C complex, whereas Chl1 is a replicative helicase; deletions were previously reported to be synthetically sick with cohesin mutants in *S. cerevisiae* (Costanzo *et al.* 2010). Swi3, a subunit of a replication fork protection complex, may normally stabilize the replication fork, which could facilitate the establishment of cohesion (Ansbach *et al.* 2008). We also found that deletion of the *mhf1* gene, which was recently found to be important for DNA replication fork stabilization (Yan *et al.* 2010), showed synthetic negative growth with the *eso1*-G799D mutation. Thus, our data support the connection between cohesion establishment and DNA replication forks.

### Deletions of genes involved in heterochromatin formation show a synthetic negative interaction with eso1 acetyltransferase mutants

In *S. pombe*, Swi6p, the homolog of human heterochromatin protein1, is critical for heterochromatin formation and the recruitment of cohesin to the centromeric region. *swi6Δ* has been shown to have synthetic growth defects with cohesin mutants (Bernard *et al.* 2001; Nonaka *et al.* 2002). Interestingly, in the cells of patients with RBS, cohesion disruption is specifically found at heterochromatin regions, further indicating a potential physical and/or genetic interaction between cohesin and heterochromatin in mammalian cells.

**Figure 2:**
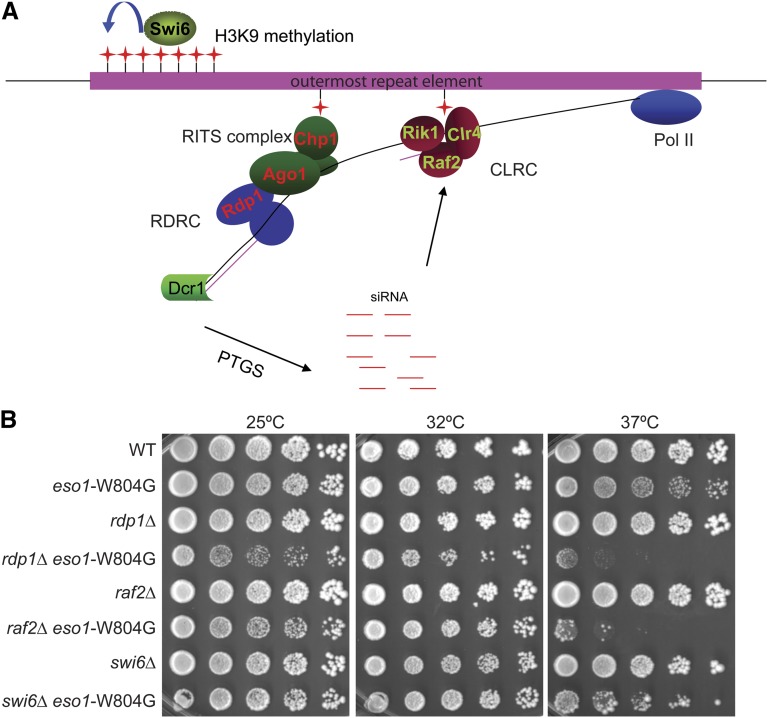
*eso1*-W804G showed negative genetic interaction with *rdp1*Δ, *raf2*Δ, and *swi6*Δ. (A) Illustration depicting the role of various proteins in heterochromatin formation. (B) The *eso1*-W804G mutant alone grew normally at 32° and was only slightly sick at 37°. However, combining the *eso1*-W804G mutation with *rdp1*, *raf2*, or *swi6* deletion made the cells grow poorly at 37°.

In our study, we found that the *eso1*-G799D mutation showed a synthetic negative effect not only with *swi6Δ* but several additional genes involved in heterochromatin formation. To further confirm the genetic interaction between *eso1* and genes participating in heterochromatin formation, we combined the *eso1*-W804G mutation with *rdp1Δ*, *raf2Δ*, and *swi6Δ*. These genes contribute to heterochromatin formation in different ways. Rdp1p, an RNA-dependent RNA polymerase, is a component of RNA-dependent RNA polymerase complex, which facilitates the methylation of H3K9 by CLRC (containing Raf2). Rdp1 is not in our synthetic sick table (Table S2) because of the data processing to eliminate linkage biases. After H3K9 methylation, Swi6 can be recruited, and heterochromatin is established (Grewal and Jia 2007; Reddy *et al.* 2011). We found that all three double mutants grew poorly at 37° (Figure 2B). Interestingly, the growth defect with *rdp1Δ* and *raf2Δ* is even stronger than that observed with *swi6Δ*, suggesting that disrupting the function of these complexes in heterochromatin formation has a stronger effect than *swi6Δ*.

Given the genetic interaction between *eso1* and heterochromatin formation genes, we asked whether the *eso1* mutations would affect gene silencing at heterochromatin regions. To address this question, we used strains containing *ura4+* inserted at pericentric (imr1R::*ura4+*, otr1R::*ura4+*) and mating type(mat3::*ura4+*) heterochromatin regions. The silencing of *ura4+* expression at heterochromatin region enables cell growth on plates with 5-fluoroorotic (FOA), which is toxic to cells with *ura4+* expression. As a control we deleted *rik1*, which has previously been reported to disrupt heterochromatin in these regions. Rik1p functions at an early step in heterochromatin formation, as it is required for proper H3K9 methylation (Partridge *et al.* 2002) and Swi6 localization (Ekwall *et al.* 1996). This deletion made the cells unable to grow on FOA plates as previously reported [(Hong *et al.* 2005) Figure S2]. Strains with either the *eso1*-G799D or *eso1*-W804G mutation can still grow on the plates containing FOA, indicating that *eso1* mutations do not have significant effects on the silencing of the *ura4+* reporter gene at the outermost centromeric repeats, innermost centromeric repeats or mating type region. Thus, the acetyltransferase activity of Eso1p does not appear to contribute to silencing at these regions of heterochromatin in *S. pombe*.

### A genetic screen with the mis4-242 allele

To distinguish whether the synthetic negative interacted genes were specific to *eso1*, we crossed the *mis4*-242 PEM2 strain against the whole-gene deletion collection from BIONEER and followed the same procedure used for *eso1*-G799D genetic screen. We found 92 gene deletions that showed a significant negative interaction with the *mis4*-242 mutant by statistical analysis (adjusted *P* < 0.05 and average difference of colony size >25; Table S3). Gene Ontology (GO) term analysis was conducted, and several GO categories are highly overrepresented in our synthetic sick gene list. Consistent with the fundamental role of *mis4* in cohesin loading onto chromatin, GO terms such as mitotic cell cycle, sister chromatid cohesion, and response to DNA damage stimulus were highly overrepresented (Table S5). Again we found the GO category “gene silencing” was overrepresented. This result argues for a general connection between heterochromatin formation and cohesin rather than any specific connection between *eso1* and heterochromatin. Interestingly, we found all checkpoint clamp complex gene deletions (*rad1*, *hus1*, *rad9*) showed negative synthetic interaction with *mis4*-242.

**Figure 3:**
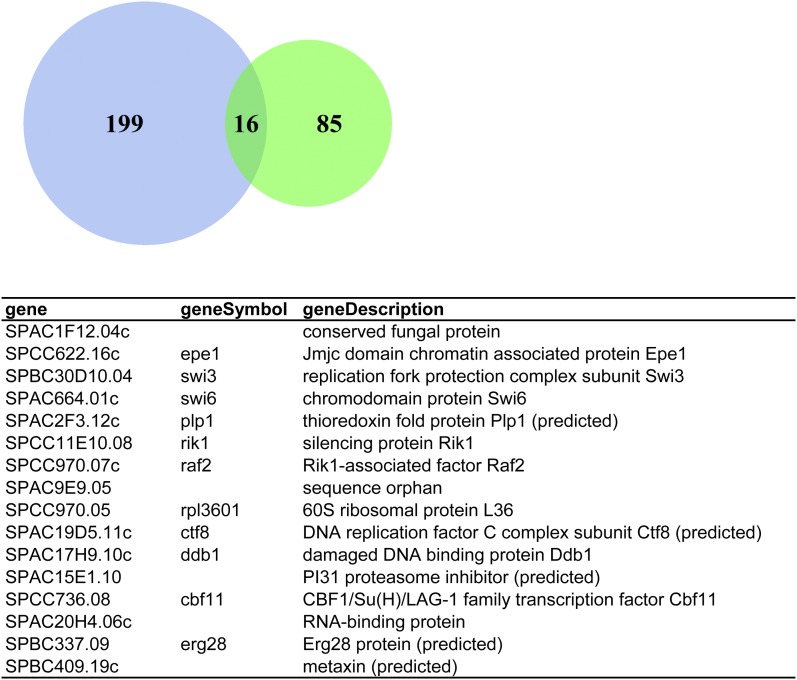
Intersection of *mis4* (green) and *eso1* (blue) screen and table of shared negative interactors

We found 16 gene deletions that shared negative synthetic growth interaction with both *eso1*-G799D and *mis4*-242 mutants (Figure 3). Four of these were heterochromatin genes: *swi6*, *clr3*, *raf2*, and *epe1*. In addition, genes important for DNA replication, such as *ctf8* and *swi3* also show synthetic negative interactions with both *eso1* and *mis4*, which is consistent with the connection between cohesin and DNA replication. Interestingly, one of the genes encodes a protein from the 60S subunit of the ribosome. It has been recently proposed that mutations in cohesin genes may impair ribosome biogenesis by compromising nucleolar structure and function (Bose *et al.* 2012; Gard *et al.* 2009). Deletions of some ribosomal protein genes also have been shown to be synthetically sick with cohesin mutants in *S. cerevisiae* (Costanzo *et al.* 2010).

### Deletion of chromatin effectors Spt2, Not3, and Rox3 can partially rescue the growth of the eso1-G799D mutant strain

**Figure 4:**
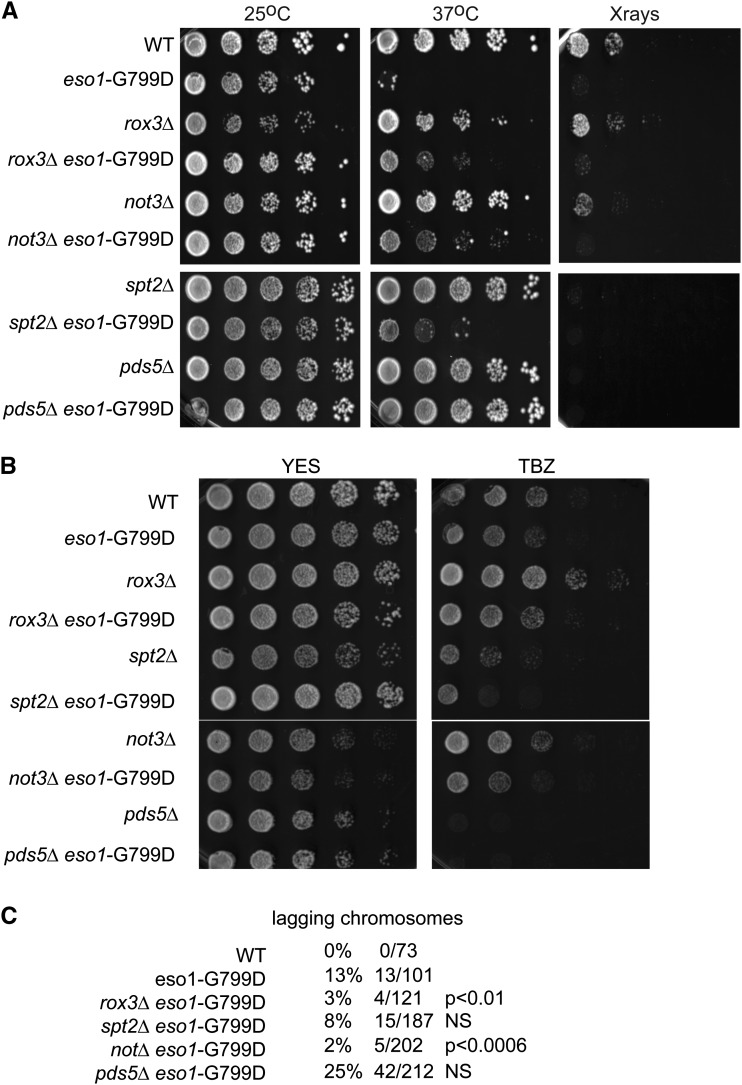
Deletions that rescue the *eso1*-G799D allele at nonpermissive temperature. Deletion of *rox3*, *not3*, and *spt2* can partially rescue the growth defect at 37° of *eso1*-G799D mutation. (A) *rox3*, *spt2*, and *not3* deletion can partially rescue the growth defect at 37° caused by *eso1*-G799D mutation. *pds5* deletion was used as the control. None of these deletions can alleviate the hypersensitivity of the *eso1*-G799D mutant to 75Gy X-ray treatment. Deletions that rescue the *eso1*-G799D allele at nonpermissive temperature do not rescue TBZ sensitivity (B), but show partial rescue of lagging chromosomes (C).

Taking advantage of the growth defect at 37° of *eso1*-G799D mutant, we were able to screen for suppressors of the temperature sensitive phenotype by growing the double mutants at 25° and 37°. Most of the double mutants can only grow at 25° but not 37° as predicted. Consistent with published data, we found that deletion of either *pds5Δ* or *wpl1Δ* can strongly rescue the growth defect of *eso1*-G799D mutant at 37°(Tanaka *et al.* 2001; Feytout *et al.* 2011). Besides *pds5Δ* and *wpl1Δ*, we also identified that *spt2Δ*, *not3*Δ, or *rox3Δ/med19*Δ can partially rescue the *eso1*-G799D mutant (Figure 4). Spt2 is an HMG-like nonhistone chromatin component, which is involved in gene regulation by affecting transcription initiation, elongation and polyadenylation (Perez-Martin and Johnson 1998; Hershkovits *et al.* 2006; Nourani *et al.* 2006). Not3 is a component of the CCR4-NOT complex, an evolutionarily conserved global transcriptional regulator (Liu *et al.* 1998; Chen *et al.* 2001). Rox3 was identified as a subunit of the evolutionarily conserved RNA pol II mediator complex (Spahr *et al.* 2001), which is composed of four modules (head, middle, tail, and kinase). Rox3 belongs to the head module. Deletion of *rox3* in *S. cerevisiae* has previously been to shown to release the middle module of mediator. The net effect is that the complex can no longer function as a conduit between activators and the core transcription machinery (Baidoobonso *et al.* 2007).

Because the *eso1*-G799D mutant is hypersensitive to X-ray treatment, we treated the double mutants containing both *eso1*-G799D mutation and *rox3Δ*, *spt2Δ*, and *not3Δ* deletion with X-rays. We found that none of these gene deletions could rescue the growth defect caused by *eso1*-G799D mutation after 75Gy X-ray treatment (Figure 4). Moreover, *pds5Δ* or *spt2Δ* alone made the cells hypersensitive to X-rays.

Next we checked rescue of TBZ sensitivity (Figure 4B). We found that *rox3Δ* alone made cells more resistant to TBZ, *spt2Δ*, or *pds5Δ* made cells more sensitive to TBZ, and *not3Δ* showed no significant effect. None of the double mutants showed increased tolerance to TBZ. We also checked the frequency of lagging chromosome in double mutants containing both *eso1*-G799D and *spt2Δ*, *not3*Δ, *rox3Δ*, or *pds5Δ* deletion. We found partial rescue of lagging chromosomes by deletion of *rox3Δ* (3%, *P* < 0.01), or *not3Δ* (2%, *P* < 0.0006), whereas *spt2Δ* was not statistically significant although it showed some rescue (8%). The *P* values, derived from the Fisher test, are relative to *eso1*-G799D (13%). The partial rescue of lagging chromosomes in the *eso1*-G799D background might contribute to the partial rescue of the growth defect of the double mutants at 37°. However, *pds5Δ* can fully rescue the growth of *eso1*-G799D mutant at 37° but does not rescue the lagging chromosome defect. This finding suggests that the chromatin effectors may partially rescue the cohesion defect, but interestingly, the growth rescue by *pds5Δ* does not include cohesion rescue.

## Discussion

In the present study, we carried out a genetic screen to explore the interactions between cohesin and nonessential genes in *S. pombe*. We found that deletions of genes associated with heterochromatin showed significant negative effects with *eso1* and *mis4* mutations. In *S. pombe*, it has been proposed that Swi6p directly recruits cohesin (Nonaka *et al.* 2002). Although deletion of *swi6* has been previously identified as having a synthetic negative effect with cohesin mutants, we have extended these finding to include subunits of the CLRC and RDRC complexes, which function in different aspects of heterochromatin function and have even stronger synthetic effects on growth. Thus, deletions that compromise heterochromatin in combination with a defect in cohesion establishment result in a significant synthetic negative phenotype. We speculate that the heterochromatin mutants negatively affect cohesion establishment at the pericentric regions, which can lead to defects in chromosome segregation. Because the *eso1* mutants do not relieve silencing at the pericentric regions, we speculate that the heterochromatic puffing associated with chromosomes in RBS may not be associated with lack of silencing. Deletions that affect DNA replication also resulted in reduced growth when combined with the *eso1* or *mis4* mutation, as has been previously observed for cohesin mutations in *S. cerevisiae* (Costanzo *et al.* 2010).

Although genes involved in DNA replication and heterochromatin were common negative interacting genes with both *eso1* -G799D and *mis4*-242, there were also some genetic interactions unique to each mutation. This finding likely reflects the different roles these genes play in cohesion, because Mis4 is a loader and Eso1 is an establishment factor. The differences in genetic interactions also may reflect additional roles of these genes. For instance, Eso1 may have acetylation targets in addition to Psm3 and Rad21 (Ghosh *et al.* 2012). Mis4 has been proposed to recruit acetyltransferases to specific gene promoters to influence their transcription (Jahnke *et al.* 2008).

Taking advantage of the temperature sensitive phenotype of the *eso1*-G799D mutant, we identified several gene deletions that allowed rescue at nonpermissive temperature. Although the cohesion antiestablishment factors were expected (*wpl1* and *pds5*), deletions that affect chromatin and transcriptional processes were unexpected (*not3*, *rox3*, *spt2*). Consistent with this observation, deletion of *SPT2* in *S. cerevisiae* has been reported to have a synthetic positive effect on growth of a *smc3-1* cohesin mutant (Costanzo *et al.* 2010). The chromatin rescuers all appear to have positive effects on cohesion function. Overall, this screen indicates that cohesin and chromatin are genetically connected through many different chromatin effectors.

## Supplementary Material

Supporting Information
