# Supplementary material for: A Genetic Screen to Discover Pathways Affecting Cohesin Function in Schizosaccharomyces pombe Identifies Chromatin Effectors
Source: G3 (Bethesda). 2012 Oct 1;2(10):1161–8. doi: 10.1534/g3.112.003327 (PMC3464108; doi:10.1534/g3.112.003327)
Supplement: Supporting Information [file supp_2_10_1161__index.html]

Supporting Information 

# A Genetic Screen to Discover Pathways Affecting Cohesin Function in *Schizosaccharomyces pombe* Identifies Chromatin Effectors

## Supporting Information for Chen *et al.*, 2012

**Files in this Data Supplement:**

- Supporting Information - Figures S1 and S2 and Tables S1-S6 (PDF, 2.4 MB)
- Figure S1 - Growth assays for 22 gene deletions which shows synthetic negative growth with *eso1*-G799D mutant (PDF, 713 KB)
- Figure S2 - *eso1* mutation has no effect on silencing of a reporter gene at centromeric and mating type heterochromatin regions (PDF, 1.2 MB)
- Table S2 - 22 genes with synthetic negative interaction with *eso1*-G799D confirmed by dilution analysis (PDF, 225 KB)
- Table S4 - GO term analysis of the genes deletion which showed negative synthetic effect with *eso1*-G799D mutant (PDF, 223 KB)
- Table S5 - GO term analysis of the genes deletion of which showed negative synthetic effect with *mis4*-242 mutant (PDF, 244 KB)
- Table S1 - Results of *eso1*-G799D screen (.xls, 47 KB)
- Table S3 - Results of *mis4*-242 screen (.xls, 26 KB)
- Table S6 - Strains (.xls, 28 KB)
