# Supplementary material for: A Genetic Screen to Discover Pathways Affecting Cohesin Function in Schizosaccharomyces pombe Identifies Chromatin Effectors
Source: G3 (Bethesda). 2012 Oct 1;2(10):1161–8. doi: 10.1534/g3.112.003327 (PMC3464108; doi:10.1534/g3.112.003327)
Supplement: Supporting Information [file supp_2.10.1161_003327SI.pdf]

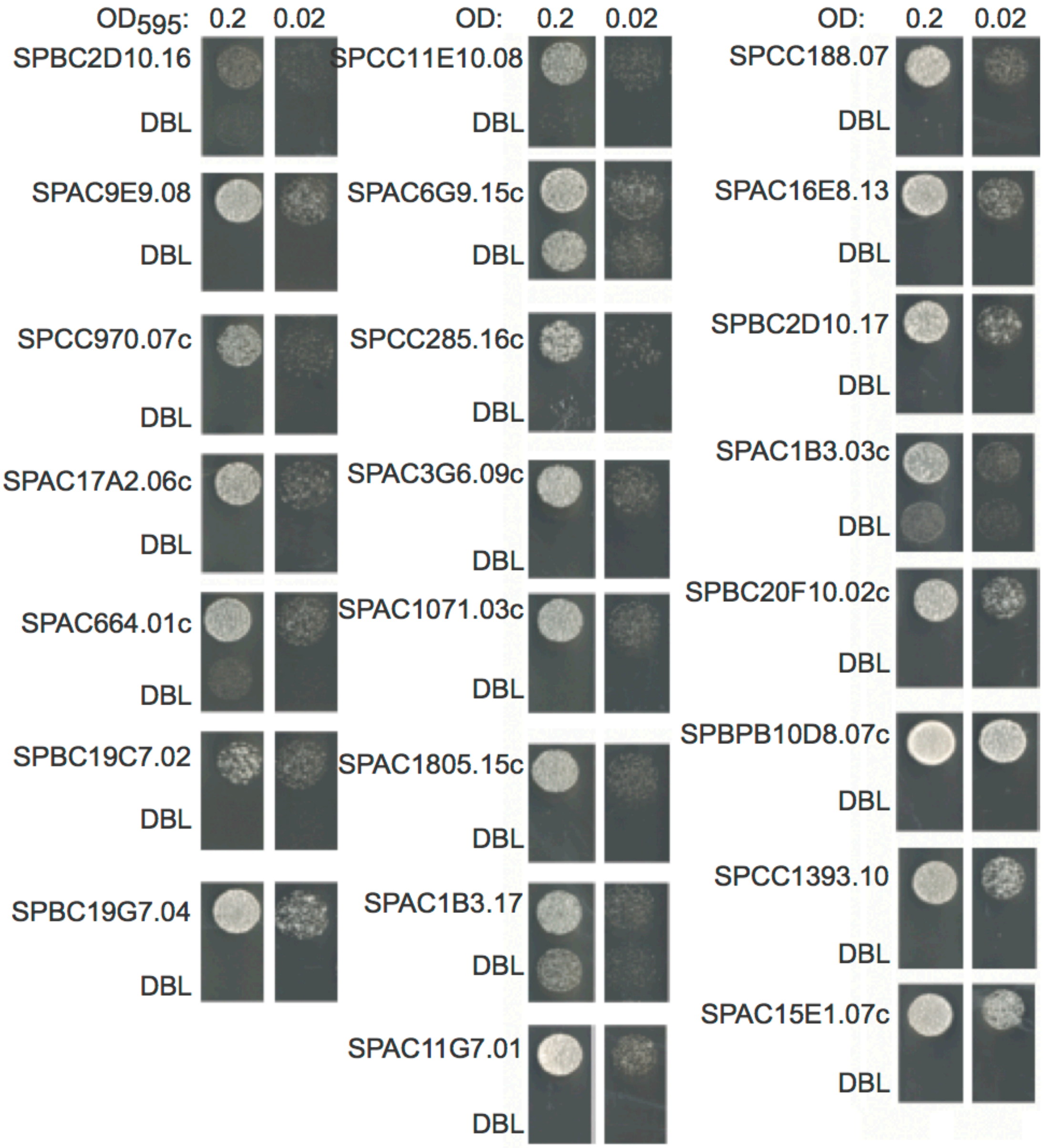

Figure S1 Growth assays for 22 gene deletions which shows synthetic negative growth with *eso1*-G799D mutant.

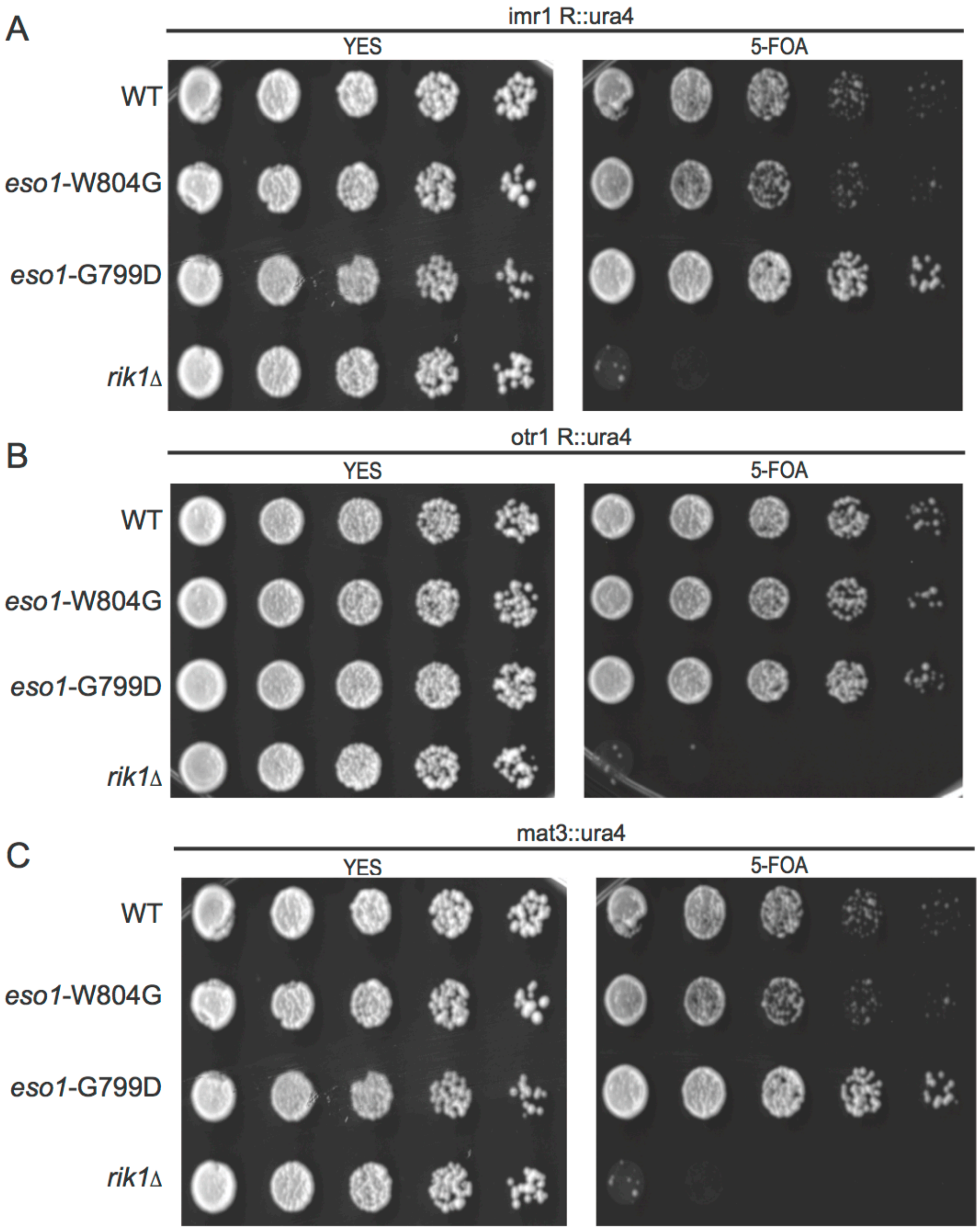

Figure S2 *eso1* mutation has no effect on silencing of a reporter gene at centromeric and mating type heterochromatin regions. Neither *eso1*-G799D nor *eso1*-W804G mutation has an effect on silencing of *ura4+* reporter gene inserted at (A) inner centromere, (B) outer centromere or (C) mating type heterochromatin regions. The strain with a deletion in *rik1* serves as a control for lack of silencing.

Table S1   Results of *eso1*-G799D screen

Available for download at http://www.g3journal.org/lookup/suppl/doi:10.1534/g3.112.003327/-/DC1.

Table S2 22 genes with synthetic negative interaction with *eso1*-G799D confirmed by dilution analysis.

gene	geneDescription
SPBC20F10.02c	DUF1741 family protein
SPCC285.16c	MutS protein homolog
SPAC664.01c	chromodomain protein Swi6
SPAC11G7.01	sequence orphan
SPAC17A2.06c	WD repeat protein Vps8 (predicted)
SPBC19C7.02	N-end-recognizing protein Ubr1
SPBC19G7.04	HMG box protein
SPCC11E10.08	silencing protein Rik1
SPCC970.07c	Rik1-associated factor Raf2
SPBC2D10.16	FANCM-MHF complex subunit Mhf1
SPAC16E8.13	ubiquitin-protein ligase E3 (predicted)
SPBC2D10.17	cryptic loci regulator Clr1
SPAC9E9.08	ATRIP, ATR checkpoint kinase regulatory subunit Rad26
SPAC1805.15c	HECT-type ubiquitin-protein ligase Pub2
SPCC1393.10	copper transporter complex subunit Ctr4
SPAC1B3.03c	cyclophilin family peptidyl-prolyl cis-trans isomerase Wis2
SPAC3G6.09c	trehalose-phosphate synthase Tps2 (predicted)
SPAC6G9.15c	sequence orphan
SPAC1B3.17	chromatin silencing protein Clr2
SPAC1071.03c	sequence orphan
SPBPB10D8.07c	membrane transporter (predicted)
SPCC188.07	telomere maintenance protein Ccq1
SPAC15E1.07c	meiotic cohesin complex associated protein Moa1

Table S3 Results of *mis4*-242 screen

Available for download at http://www.g3journal.org/lookup/suppl/doi:10.1534/g3.112.003327/-/DC1.

Table S4 GO term analysis of the genes deletion which showed negative synthetic effect with *eso1*-G799D mutant

Term Acc	Term Name	Enrich	Adj P
GO:0016458	gene silencing	OVER	4.55E-06
GO:0048523	negative regulation of cellular process	OVER	9.05E-06
GO:0007059	chromosome segregation	OVER	2.17E-05
GO:0009892	negative regulation of metabolic process	OVER	2.62E-05
GO:0022402	cell cycle process	OVER	2.57E-04
GO:0007049	cell cycle	OVER	9.44E-04
GO:0019222	regulation of metabolic process	OVER	4.90E-03
GO:0050789	regulation of biological process	OVER	5.45E-03
GO:0050794	regulation of cellular process	OVER	1.05E-02
GO:0043233	organelle lumen	OVER	1.22E-02
GO:0009893	positive regulation of metabolic process	OVER	1.27E-02
GO:0043170	macromolecule metabolic process	OVER	1.85E-02
GO:0009058	biosynthetic process	OVER	1.85E-02
GO:0043228	non-membrane-bounded organelle	OVER	2.33E-02
GO:0033036	macromolecule localization	OVER	2.37E-02
GO:0051641	cellular localization	OVER	3.45E-02
GO:0043234	protein complex	OVER	5.62E-02
GO:0016043	cellular component organization	OVER	6.36E-02
GO:0044238	primary metabolic process	OVER	8.36E-02
GO:0048522	positive regulation of cellular process	OVER	8.56E-02
GO:0006807	nitrogen compound metabolic process	OVER	8.56E-02
GO:0071841	cellular component organization or biogenesis at cellular level	OVER	9.22E-02
GO:0044446	intracellular organelle part	OVER	9.22E-02
GO:0071821	FANCM-MHF complex	OVER	7.51E-02

Table S5 GO term analysis of the genes deletion of which showed negative synthetic effect with *mis4*-242 mutant

Term Acc	Term Name	Enrich	Adj P
GO:0000086	G2/M transition of mitotic cell cycle	OVER	2.11E-02
GO:0000278	mitotic cell cycle	OVER	7.90E-03
GO:0006139	nucleobase, nucleoside, nucleotide and nucleic acid metabolic process	OVER	2.17E-02
GO:0006342	chromatin silencing	OVER	1.51E-02
GO:0007018	microtubule-based movement	OVER	4.35E-02
GO:0007062	sister chromatid cohesion	OVER	1.07E-02
GO:0007530	sex determination	OVER	1.51E-02
GO:0007531	mating type determination	OVER	1.51E-02
GO:0007535	donor selection	OVER	4.25E-02
GO:0009890	negative regulation of biosynthetic process	OVER	9.42E-03
GO:0010467	gene expression	OVER	2.01E-02
GO:0010605	negative regulation of macromolecule metabolic process	OVER	1.37E-02
GO:0019538	protein metabolic process	OVER	4.35E-02
GO:0022403	cell cycle phase	OVER	1.07E-02
GO:0031323	regulation of cellular metabolic process	OVER	1.86E-02
GO:0031324	negative regulation of cellular metabolic process	OVER	1.37E-02
GO:0044260	cellular macromolecule metabolic process	OVER	7.90E-03
GO:0045165	cell fate commitment	OVER	1.51E-02
GO:0048519	negative regulation of biological process	OVER	7.90E-03
GO:0051171	regulation of nitrogen compound metabolic process	OVER	7.90E-03
GO:0051172	negative regulation of nitrogen compound metabolic process	OVER	7.90E-03
GO:0060255	regulation of macromolecule metabolic process	OVER	1.32E-02
GO:0071842	cellular component organization at cellular level	OVER	1.86E-02
GO:0080090	regulation of primary metabolic process	OVER	1.22E-02
GO:0006974	response to DNA damage stimulus	OVER	1.37E-02

Table S6 Strains

Available for download at http://www.g3journal.org/lookup/suppl/doi:10.1534/g3.112.003327/-/DC1.
