# Supplementary material for: A Genetic Screen to Discover Pathways Affecting Cohesin Function in Schizosaccharomyces pombe Identifies Chromatin Effectors
Source: G3 (Bethesda). 2012 Oct 1;2(10):1161–8. doi: 10.1534/g3.112.003327 (PMC3464108; doi:10.1534/g3.112.003327)
Supplement: Supporting Information [file supp_2.10.1161_FigureS2.pdf]

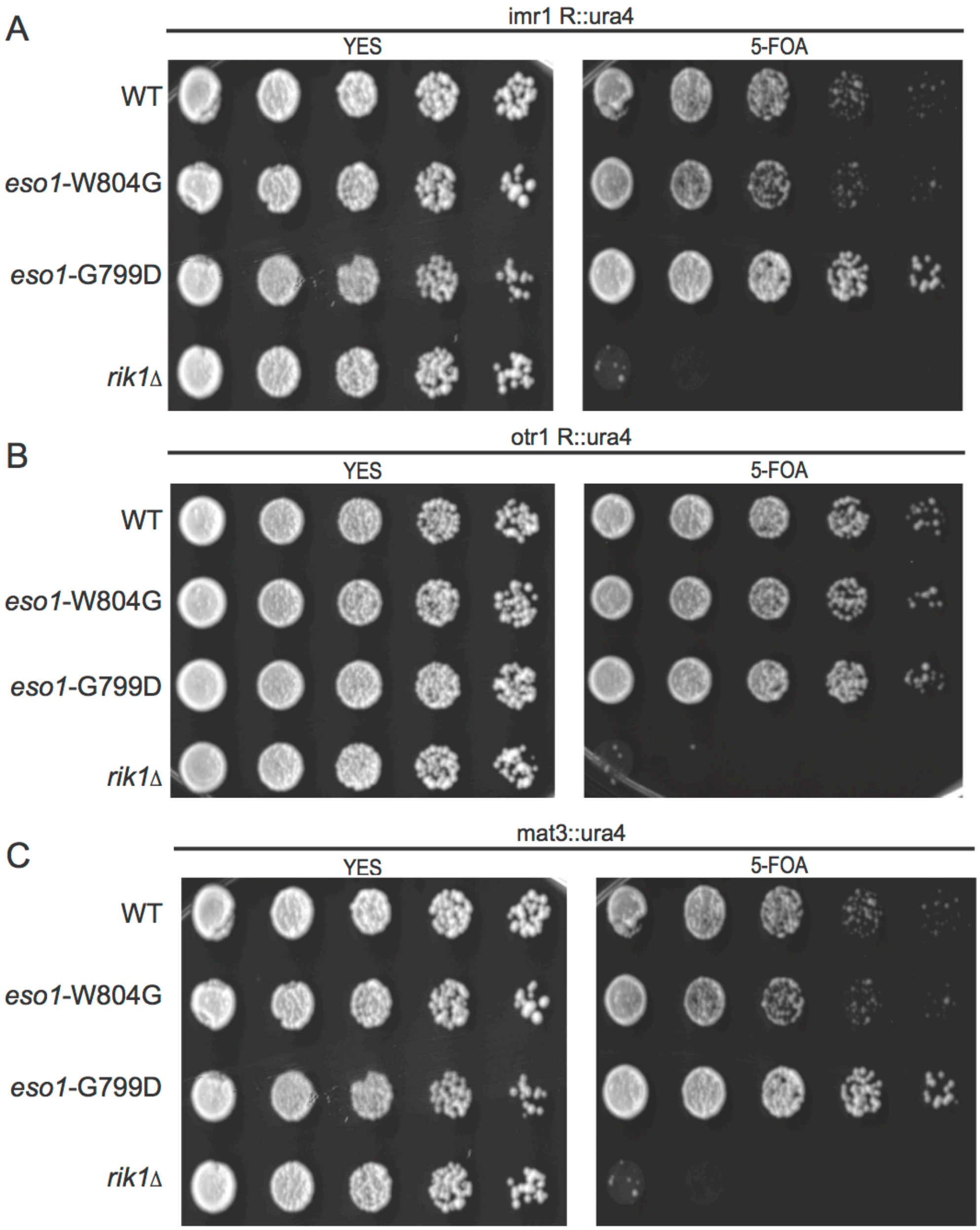

Figure S2 *eso1* mutation has no effect on silencing of a reporter gene at centromeric and mating type heterochromatin regions. Neither *eso1*-G799D nor *eso1*-W804G mutation has an effect on silencing of *ura4+* reporter gene inserted at (A) inner centromere, (B) outer centromere or (C) mating type heterochromatin regions. The strain with a deletion in *rik1* serves as a control for lack of silencing.
