# Supplementary material for: A Genetic Screen to Discover Pathways Affecting Cohesin Function in Schizosaccharomyces pombe Identifies Chromatin Effectors
Source: G3 (Bethesda). 2012 Oct 1;2(10):1161–8. doi: 10.1534/g3.112.003327 (PMC3464108; doi:10.1534/g3.112.003327)
Supplement: Supporting Information [file supp_2.10.1161_TableS2.pdf]

Table S2 22 genes with synthetic negative interaction with *eso1*-G799D confirmed by dilution analysis.

gene	geneDescription
SPBC20F10.02c	DUF1741 family protein
SPCC285.16c	MutS protein homolog
SPAC664.01c	chromodomain protein Swi6
SPAC11G7.01	sequence orphan
SPAC17A2.06c	WD repeat protein Vps8 (predicted)
SPBC19C7.02	N-end-recognizing protein Ubr1
SPBC19G7.04	HMG box protein
SPCC11E10.08	silencing protein Rik1
SPCC970.07c	Rik1-associated factor Raf2
SPBC2D10.16	FANCM-MHF complex subunit Mhf1
SPAC16E8.13	ubiquitin-protein ligase E3 (predicted)
SPBC2D10.17	cryptic loci regulator Clr1
SPAC9E9.08	ATRIP, ATR checkpoint kinase regulatory subunit Rad26
SPAC1805.15c	HECT-type ubiquitin-protein ligase Pub2
SPCC1393.10	copper transporter complex subunit Ctr4
SPAC1B3.03c	cyclophilin family peptidyl-prolyl cis-trans isomerase Wis2
SPAC3G6.09c	trehalose-phosphate synthase Tps2 (predicted)
SPAC6G9.15c	sequence orphan
SPAC1B3.17	chromatin silencing protein Clr2
SPAC1071.03c	sequence orphan
SPBPB10D8.07c	membrane transporter (predicted)
SPCC188.07	telomere maintenance protein Ccq1
SPAC15E1.07c	meiotic cohesin complex associated protein Moa1
