# Supplementary material for: A Genetic Screen to Discover Pathways Affecting Cohesin Function in Schizosaccharomyces pombe Identifies Chromatin Effectors
Source: G3 (Bethesda). 2012 Oct 1;2(10):1161–8. doi: 10.1534/g3.112.003327 (PMC3464108; doi:10.1534/g3.112.003327)
Supplement: Supporting Information [file supp_2.10.1161_TableS4.pdf]

Table S4 GO term analysis of the genes deletion which showed negative synthetic effect with *eso1*-G799D mutant

Term Acc	Term Name	Enrich	Adj P
GO:0016458	gene silencing	OVER	4.55E-06
GO:0048523	negative regulation of cellular process	OVER	9.05E-06
GO:0007059	chromosome segregation	OVER	2.17E-05
GO:0009892	negative regulation of metabolic process	OVER	2.62E-05
GO:0022402	cell cycle process	OVER	2.57E-04
GO:0007049	cell cycle	OVER	9.44E-04
GO:0019222	regulation of metabolic process	OVER	4.90E-03
GO:0050789	regulation of biological process	OVER	5.45E-03
GO:0050794	regulation of cellular process	OVER	1.05E-02
GO:0043233	organelle lumen	OVER	1.22E-02
GO:0009893	positive regulation of metabolic process	OVER	1.27E-02
GO:0043170	macromolecule metabolic process	OVER	1.85E-02
GO:0009058	biosynthetic process	OVER	1.85E-02
GO:0043228	non-membrane-bounded organelle	OVER	2.33E-02
GO:0033036	macromolecule localization	OVER	2.37E-02
GO:0051641	cellular localization	OVER	3.45E-02
GO:0043234	protein complex	OVER	5.62E-02
GO:0016043	cellular component organization	OVER	6.36E-02
GO:0044238	primary metabolic process	OVER	8.36E-02
GO:0048522	positive regulation of cellular process	OVER	8.56E-02
GO:0006807	nitrogen compound metabolic process	OVER	8.56E-02
GO:0071841	cellular component organization or biogenesis at cellular level	OVER	9.22E-02
GO:0044446	intracellular organelle part	OVER	9.22E-02
GO:0071821	FANCM-MHF complex	OVER	7.51E-02
