# Supplementary material for: A Genetic Screen to Discover Pathways Affecting Cohesin Function in Schizosaccharomyces pombe Identifies Chromatin Effectors
Source: G3 (Bethesda). 2012 Oct 1;2(10):1161–8. doi: 10.1534/g3.112.003327 (PMC3464108; doi:10.1534/g3.112.003327)
Supplement: Supporting Information [file supp_2.10.1161_TableS5.pdf]

Table S5 GO term analysis of the genes deletion of which showed negative synthetic effect with *mis4*-242 mutant

Term Acc	Term Name	Enrich	Adj P
GO:0000086	G2/M transition of mitotic cell cycle	OVER	2.11E-02
GO:0000278	mitotic cell cycle	OVER	7.90E-03
GO:0006139	nucleobase, nucleoside, nucleotide and nucleic acid metabolic process	OVER	2.17E-02
GO:0006342	chromatin silencing	OVER	1.51E-02
GO:0007018	microtubule-based movement	OVER	4.35E-02
GO:0007062	sister chromatid cohesion	OVER	1.07E-02
GO:0007530	sex determination	OVER	1.51E-02
GO:0007531	mating type determination	OVER	1.51E-02
GO:0007535	donor selection	OVER	4.25E-02
GO:0009890	negative regulation of biosynthetic process	OVER	9.42E-03
GO:0010467	gene expression	OVER	2.01E-02
GO:0010605	negative regulation of macromolecule metabolic process	OVER	1.37E-02
GO:0019538	protein metabolic process	OVER	4.35E-02
GO:0022403	cell cycle phase	OVER	1.07E-02
GO:0031323	regulation of cellular metabolic process	OVER	1.86E-02
GO:0031324	negative regulation of cellular metabolic process	OVER	1.37E-02
GO:0044260	cellular macromolecule metabolic process	OVER	7.90E-03
GO:0045165	cell fate commitment	OVER	1.51E-02
GO:0048519	negative regulation of biological process	OVER	7.90E-03
GO:0051171	regulation of nitrogen compound metabolic process	OVER	7.90E-03
GO:0051172	negative regulation of nitrogen compound metabolic process	OVER	7.90E-03
GO:0060255	regulation of macromolecule metabolic process	OVER	1.32E-02
GO:0071842	cellular component organization at cellular level	OVER	1.86E-02
GO:0080090	regulation of primary metabolic process	OVER	1.22E-02
GO:0006974	response to DNA damage stimulus	OVER	1.37E-02
