# Supplementary material for: A Genetic Screen to Discover Pathways Affecting Cohesin Function in Schizosaccharomyces pombe Identifies Chromatin Effectors
Source: G3 (Bethesda). 2012 Oct 1;2(10):1161–8. doi: 10.1534/g3.112.003327 (PMC3464108; doi:10.1534/g3.112.003327)
Supplement: Supporting Information [file supp_2.10.1161_FigureS1.pdf]

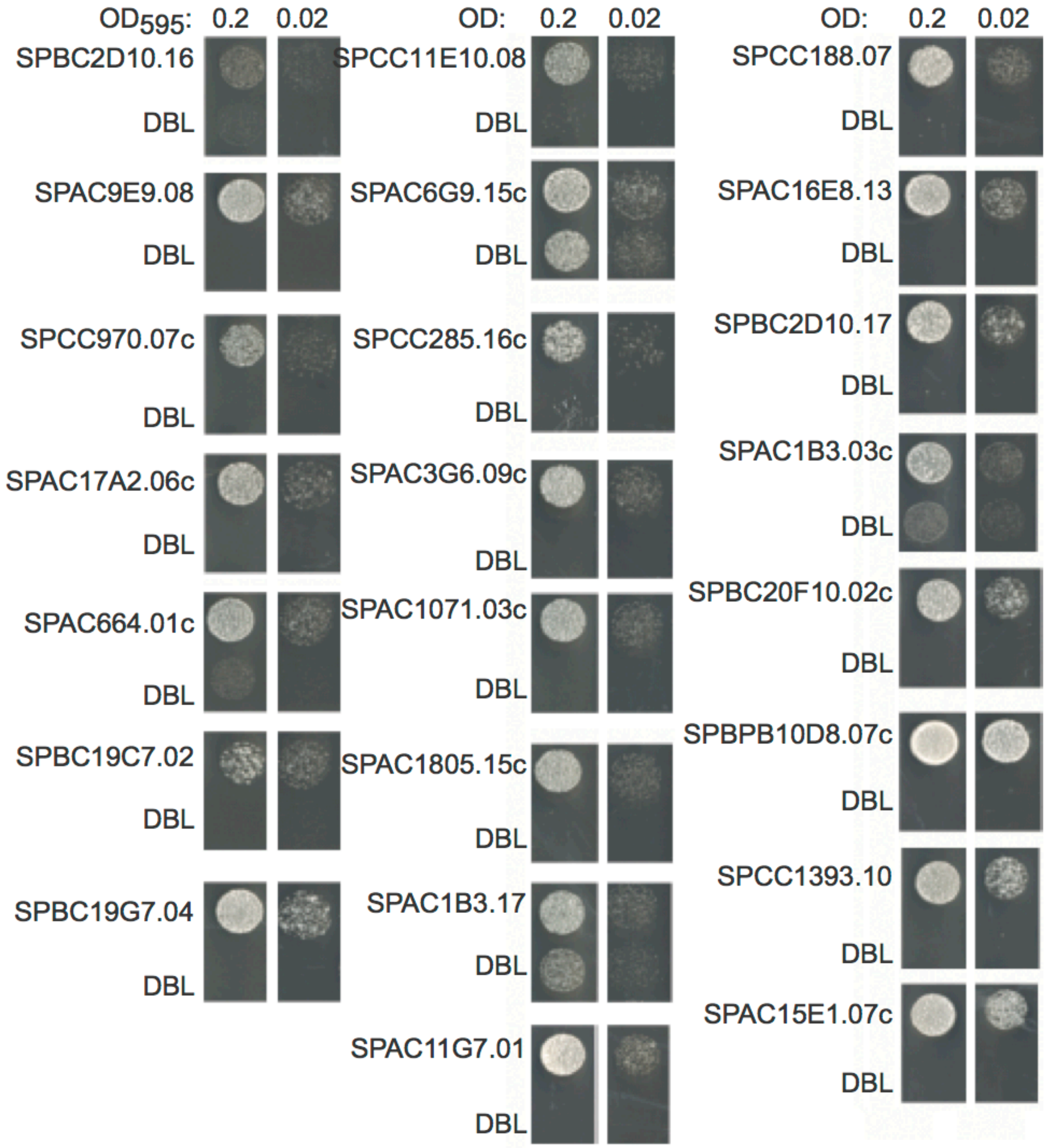

Figure S1 Growth assays for 22 gene deletions which shows synthetic negative growth with *eso1*-G799D mutant.
